# Effects of an APOE Promoter Polymorphism on Fronto-Parietal Functional Connectivity During Nondemented Aging

**DOI:** 10.3389/fnagi.2020.00183

**Published:** 2020-06-30

**Authors:** Qirui Zhang, Lingli Wu, Chao Du, Kai Xu, Jinping Sun, Junying Zhang, He Li, Xin Li

**Affiliations:** ^1^Institute of Criminology, People’s Public Security University of China, Beijing, China; ^2^State Key Laboratory of Cognitive Neuroscience and Learning and IDG/McGovern Institute for Brain Research, Beijing Normal University, Beijing, China; ^3^BABRI Centre, Beijing Normal University, Beijing, China; ^4^The Affiliated Hospital of Qingdao University, Shandong, China

**Keywords:** APOE promoter, brain connectome, fronto-parietal network, working memory, aging

## Abstract

**Background**: The rs405509 polymorphism ofthe apolipoprotein E (APOE) promoter is related to Alzheimer’sdisease (AD). The T/T allele of rs405509 is known to decrease the transcription of the APOE gene and lead to impairments in specific brain structural networks with aging; thus, it is an important risk factor for AD. However, it remains unknown whether rs405509 affects brain functional connectivity (FC) in aging.

**Methods**: We investigated the effect of the rs405509 genotype (T/T vs. G-allele) on age-related brain FC using functional magnetic resonance imaging. Forty-five elderly TT carriers and 45 elderly G-allele carriers were scanned during a working memory (WM) task.

**Results**: We found that TT carriers showed an accelerated age-related increase in functional activation in the left postcentral gyrus compared with G-allele carriers. Furthermore, the FC between the left postcentral gyrus and some key regions during WM performance, including the right caudal and superior frontal sulcus (SFS), was differentially modulated by age across rs405509 genotype groups.

**Conclusions**: These results demonstrate that the rs405509 T/T allele of APOE causes an age-related brain functional decline in nondemented elderly people, which may be beneficial for understanding the neural mechanisms of rs405509-related cognitive aging and AD pathogenesis.

## Introduction

As the most common cause of dementia in the world, Alzheimer’s disease (AD) has been studied for many years (Mondadori et al., 2007). To date, the specific pathophysiology of AD remains unclear. Even though there is a wide range of different opinions, prior research has shown the importance of genetic factors (van Duijn et al., 1991). Apolipoprotein E4 (APOE4) is deemed to be a genetic risk factor for sporadic AD (Strittmatter et al., 1993). Compared with noncarriers, carriers of APOE4 not only have a higher risk of developing AD but also suffer from poorer memory (Corder et al., 1993). However, more research is needed to clarify the underlying mechanisms.

Currently, there is no unified conclusion about how the APOE4 genotype will affect the brain structure before mild cognitive impairment (MCI) or AD has developed (Bookheimer and Burggren, 2009). Some studies have suggested structural differences. For example, among elderly individuals, APOE4 carriers showed a smaller hippocampus than noncarriers (den Heijer et al., 2002), and even in the child and adolescent stages, APOE4 carriers showed a thinner entorhinal cortex than noncarriers (Shaw et al., 2007). However, other studies found no significant difference between the two groups in the middle temporal lobe (Lemaítre et al., 2005; Fennema-Notestine et al., 2011). Moreover, there was higher activation in the frontal and parietal lobes in elderly APOE4 carriers than noncarriers (Wishart et al., 2006; Mondadori et al., 2007).

It is important to note that the e4 allele is only one of the AD-related APOE polymorphisms. Reliable evidence suggests that polymorphisms within the APOE promoter region significantly affect AD occurrence (Lambert et al., 1998b; Bertram et al., 2007; Lescai et al., 2011). Among these polymorphisms, rs405509 has shown the most important role in neural APOE gene expression and was also associated with an excess risk for AD, in which the T/T allele was most at risk (Lambert et al., 1998a). Our recent studies found that the rs405509 genotype modulated cortical thickness in the parahippocampus and affect the work of white matter structural networks in the posterior cingulate cortices during nondemented aging (Chen et al., 2015; Shu et al., 2015). Disconnections in brain structure in TT carriers may induce dysfunction. This may be the neural basis for developing cognitive impairments in TT carriers.

Working memory (WM) is vulnerable in AD patients, and it is also a fundamental cognitive function sensitive to aging (Belleville et al., 2007, 2008). Meanwhile, alterations in functional connectivity (FC) are independent of early brain functional changes, which are related to impaired cognitive ability (Sperling et al., 2010; Cai et al., 2017). The panoply of cognitive functions that underpin everyday human experience requires precisely choreographed patterns of interaction among networked brain regions (Leech et al., 2011). The FC could integrate ongoing information from anatomically separate brain regions very efficiently. Thus, FC is considered as a good indicator for cognitive performance. Previous studies have used FC in task-related networks known to be involved in cognitive functions as a predictor of memory performance (Smith et al., 2009; Li et al., 2015). The identification of brain functional activation and connection patterns during WM tasks can potentially enhance our understanding of subtle brain dysfunction in elderly individuals with AD risk factors.

We conducted an fMRI study in subjects performing a visual 2-back and 1-back WM task to determine whether the TT genotype could influence neural structure or function during nondemented aging. Here, we also drew a map of the number of connections and the strength of FC between hotspots determined by a meta-analysis of brain activation observed during WM task performance (Nee et al., 2013). To clarify the polymorphism’s influence on neural systems, it is important to include nondemented aging individuals, which may help us to illustrate the modulatory effect of the polymorphism on the risk of developing AD.

### Participants

This study included 90 right-handed, native Chinese participants. All data were from the Beijing Aging Brain Rejuvenation Initiative (BABRI). All of the subjects met the following criteria: (1) a score of at least 24 on the Mini-Mental Status Examination-Chinese version (MMSE; Anderson, 2015); (2) age older than 50 years; (3) 6 or more years of education (Peluso et al., 2015); (4) no history of neurologic, psychiatric, or systemic disease; (5) no history of addiction or psychoactive medication use; (6) no conditions that would have influenced cerebral function including serious vascular diseases, head trauma, tumor, current depression, alcoholism, and epilepsy; and (7) able to cope with the physical demands of the MRI scanning. Subjects with the following conditions were excluded from this study (Peluso et al., 2015): (1) structural abnormalities other than cerebrovascular lesions, such as tumors, subdural hemeatomas, and contusions because of previous head trauma, that could impair cognitive function; (2) large vessel disease, such as cortical or subcortical infarcts and watershed infarcts; and (3) diseases with WM lesions, such as normal-pressure hydrocephalus and multiple sclerosis. The study was approved by the ethics committee and institutional review board (IRB) of Beijing Normal University Imaging Center for Brain Research, and written informed consent was provided by each participant.

### Neuropsychological Testing

At the screening visit, all participants received a battery of neuropsychological tests to assess general mental status and other cognitive domains, including episodic memory, processing speed, visual–spatial ability, executive function, and language ability. The Chinese translation of the MMSE served as a general cognitive function test. The other tests included the following: (1) episodic memory, Auditory Verbal Learning Test (AVLT; Markowitz, 2015) and Rey–Osterrieth Complex Figure test (ROCF; recall component; Peluso et al., 2015); (2) processing speed, Symbol Digit Modalities Test (SDMT; Markowitz, 2015); (3) visual–spatial ability, ROCF (Ávila et al., 2015); (4) language ability, Category Verbal Fluency Test (CVFT) (Jeon and Han, 2012); and (5) executive function, Stroop Color–Word Test (Ryan et al., 2015).

### Analysis of Genotyping

Genomic DNA was extracted from the blood samples of the subjects using the standard method (Guha et al., 2018). The participants were pre-screened for APOE genotype using a TaqMan single nucleotide polymorphism (SNP) genotyping assay on a 7900 HT Fast Real-Time PCR system (Applied Biosystems, Foster City, CA, USA; Seripa et al., 2011). Briefly, two SNPs, rs429358 and rs7412, collectively identified the APOE genotype (with the haplotype of rs429358-rs7412: T/T identified the ε2 variant, the haplotype of rs429358-rs7412: G/T identified the ε3 variant, and the haplotype of rs429358-rs7412: G/G identified the ε4 variant). All participants were genotyped for one SNP in the APOE promoter region (rs405509) using previously published methods (Moraes et al., 2012). Genotype identifications were manually and independently verified by two laboratory personnel. The sample success rates for all the three polymorphisms were 100%, and the reproducibility of the genotyping was 100% according to a duplication analysis of at least 10% of the genotypes. There were 45 rs405509 T/T carriers and 45 rs405509 GG/GT carriers included in our present study.

### Experimental Paradigm

Participants was tested by an n-back task which has a blocked periodic design that incorporated alternating 0-back, 1-back, and 2-back three conditions to assess their WM. At the start of each block, participants studied the task instructions; then, we presented a fixation cross lasting 10 s. After that, white numbers (0–9) were continuously presented on the black background in pseudorandom order for 1,000 ms each, following a same blank interval for 1,000 ms. During the 0-back load task, participants were asked to respond to the target number “1.” In the 1-back load condition, participants should respond once the current number matches the number just presented one trial before. In the 2-back load condition, the target was changed to be the number presented two trials ago. There were nine task blocks in total, including three conditions presenting three times each. In each block, a half of trails was the target trials. All stimuli were presented by E-Prime 1.2 software (Psychology Software Tools Inc., Sharpsburg, PA, USA). Participants made their responses by pressing the button on MRI-compatible response box.

### MRI Data Acquisition

Magnetic resonance imaging data acquisition was performed using a Siemens Trio 3.0 Tesla scanner (Trio; Siemens, Erlangen, Germany) in the Imaging Center for Brain Research at Beijing Normal University. Participants lay supine with their head snugly fixed by straps and foam pads to minimize head movement. The functional images were acquired using an echo-planar imaging sequence: 33 axial slices, repetition time (TR) = 2,000 ms, echo time (TE) = 30 ms, slice thickness = 3.5 mm, flip angle = 90°, field of view (FOV) = 200 mm * 200 mm, and acquisition matrix = 64 * 64. The scan lasted for 480 s.

### Functional Image Analysis

All image preprocessing and analyses were conducted using Statistical Parametric Mapping (SPM8^1^). Preprocessing procedures included slice timing, within-subject interscan realignment to correct for possible movement, spatial normalization to the template in MNI space, resampling to 3 * 3 * 3 mm^3^, and smoothing with an 8-mm FWHM Gaussian kernel. Finally, all of the images were temporally filtered with a cutoff frequency of 0.01 Hz to eliminate high-frequency noise and low-frequency drift. At the single-subject level, data were analyzed according to the fixed effects model (SPM5). Contrast images for 1-back minus 0-back conditions and 2-back minus 0-back conditions were calculated. At the group level, one-sample *t*-tests were further performed. Statistical images were analyzed using a corrected threshold of *P* < 0.05 (AlphaSim corrected) to identify significant activation in each group. Finally, activation differences between groups were computed by a two-sample *t*-test using an inclusive regions mask. An AlphaSim-corrected threshold of *P* < 0.05 was identified as significantly activated.

We also mapped the number of connections and the strength of FC between meta-analytically determined hotspots of brain activation observed during WM tasks and then the functional connections during the N-back tasks in the two groups were analyzed for partial correlations with age.

### Statistical Analysis

The Hardy–Weinberg test was completed using PLINK software. Independent two-sample *t*-tests were used to assess between-group differences in age and education. A chi-square test was used to compare gender ratio differences and genotyping differences. For neuropsychological assessment, a linear regression analysis was used for the neuropsychological test (Cognition = age * rs405509 + rs405509 + age + education + APOE4 + gender), and then cognitive function was assessed in each genotype in a partial correlation analysis with age (gender, education, and APOE4 status were included as covariates).

We used analysis of covariance (ANCOVA) to explore the effects of rs405509 on memory-related brain activity changes and brain FC. To assess changes in FC with age in different genotypes, we also performed a partial correlation analysis of each functional connection with age using Pearson’s correlations in each group. APOE4 carrier status, age, educational level, and gender were used as covariates. The above analysis was performed in SPSS 17.0.

## Results

### Demographic and Neuropsychological Measurements

The demographic characteristics of the rs405509 TT carrier and GG/GT carrier groups are shown in Table 1. We did not find any differences in age, gender, or education between the two groups. There was a marginally significant difference in APOE4 status.

**Table 1:** Demographic characteristics of rs405509 TT carriers and GG/GT carriers.

	TT carriers (*n* = 45)	GG/GT carriers (*n* = 45)	*P*
Gender	22 M/23 F	17 M/28 F	0.288
APOE4	32−/13+	39−/6+	0.071
Age (years)	63.6 ± 7.238	62.11 ± 5.757	0.283
Education (years)	10.21 ± 2.801	10.56 ± 3.123	0.583

*APOE, apolipoprotein E*.

The results of linear regression analysis can be found in Table 2 with cognitive test outcomes as dependent variables, rs405509 genotype, age, rs405509 genotype * age (interaction item), APOE4 status, gender, and educational level as independent variables. The results showed that the interaction between rs405509 genotype and age had a significant effect on MMSE (*t* = 3.06, *p* = 0.003) and Stroop C-B (*t* = −3.036, *p* = 0.003) scores. The partial correlation analysis results are shown in Table 3. We found that there was a negative correlation between age and attention (*r* = −0.492, *p* = 0.003) and a positive correlation with executive function (*r* = 0.372, *p* = 0.028) in TT carriers.

**Table 2:** The linear regression analysis result of model and interaction item.

Cognition	Model		Age*genotype	
	*F*	*p*	*T*	*p*
MMSE	4.45	0.001	3.060	0.003
AVLT-delay recall	2.72	0.018	−0.541	0.590
AVLT-total	3.23	0.007	0.708	0.481
ROCF-delay recall	2.53	0.027	1.088	0.280
SDMT	5.41	0.000	0.801	0.426
CVFT	1.02	0.421	0.349	0.728
Stroop C-B Time(s)	4.19	0.001	−3.036	0.003

*MMSE, Mini-Mental State Examination; AVLT, Auditory Verbal Learning Test; ROCF, Rey–Osterrieth Complex Figure; SDMT, Symbol Digit Modalities Test; CVFT, Category Verbal Fluency Test; Stroop C-B, Stroop Word Color Test C-B*.

**Table 3:** The results of partial correlation analysis.

Cognition	TT carriers (*n* = 45)		GG/GT carriers (*n* = 45)	
	*r*	*p*	*r*	*p*
MMSE	−0.28	0.092	0.11	0.528
AVLT-delay recall	−0.15	0.372	−0.46	0.006
AVLT-total	−0.21	0.208	−0.27	0.120
ROCF-delay recall	−0.19	0.263	−0.30	0.079
SDMT	−0.49	0.003	−0.43	0.010
CVFT	0.34	0.046	−0.33	0.052
Stroop C-B time	0.37	0.028	−0.17	0.315

*MMSE, Mini-Mental State Examination; AVLT, Auditory Verbal Learning Test; ROCF, Rey–Osterrieth Complex Figure; SDMT, Symbol Digit Modalities Test; CVFT, Category Verbal Fluency Test; Stroop C-B, Stroop Word Color Test C-B*.

### The Effects of rs405509 on Memory-Related Brain Activity Changes

**Figure 1:**
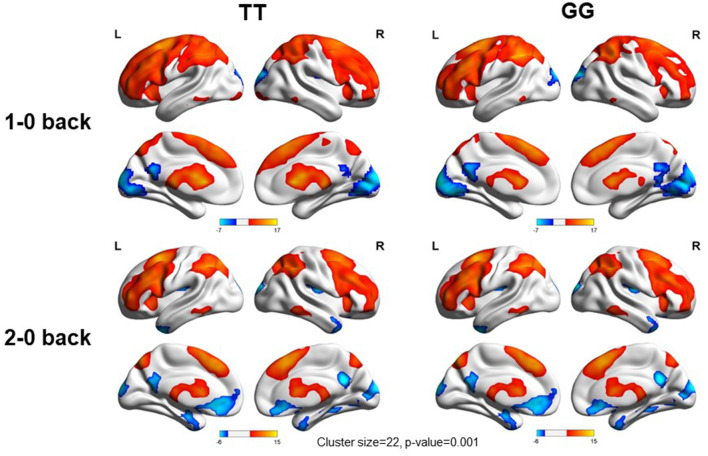
Views of activation during n-back tasks for TT and GT/GG groups displayed on a custom template sagittal brain surface created from subjects in the study. Note. L: Left, R: Right.

**Figure 2:**
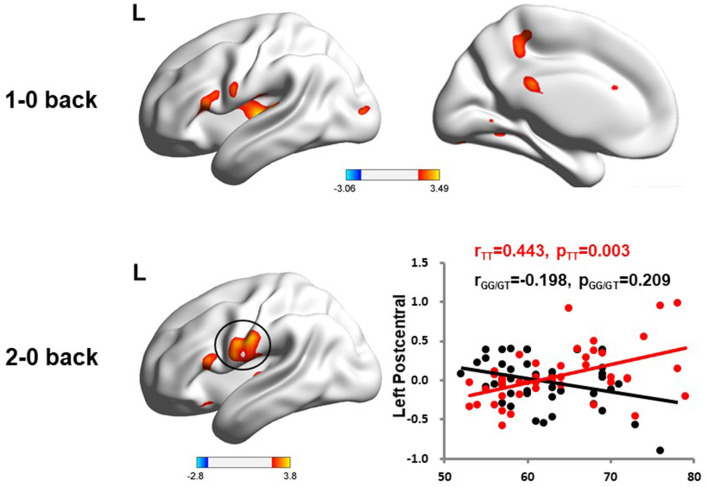
The significant interaction of age and rs405509 genotype on brain activity changes in condition 1-0 back and 2-0 back.

We used the rs405509 genotype and age interaction analysis to determine the effect of rs405509 on brain activation, in which the APOE4 status, gender, and educational level were used as covariates, and multiple comparison corrections were used (AlphaSim corrected, *p* = 0.001, cluster size = 22). In the n-back task, activation views of the TT group and the GT/GG group were displayed on the sagittal brain surface of a custom template created by subjects in this study (Figure 1). We found that the left insula and left superior temporal lobe had significant interactions with genotype and age in the 1–0 back condition and left postcentral lobe in the 2–0 back condition (Figure 2; Table 4).

**Table 4:** The significant interaction of age and rs405509 genotype on brain activity changes in condition 1-0 back and 2-0 back.

Brain regions	Cluster sizes	MNI coordinates (mm)			*T*
		*X*	*Y*	*Z*	
1–0 back					
Left insula	13	−39	−18	6	3.438
Left superior temporal gyrus	10	−54	−18	12	3.489
2–0 back					
Left postcentral gyrus	46	−63	−9	30	3.800

*Alphasim, p = 0.001, cluster sizes = 22*.

The partial correlation analysis showed that the rs405509 T/T carriers (R_TT_ = 0.443, P_TT_ = 0.003) showed an accelerated age-related increase in functional activation in the left postcentral region (or precentral) compared with G-allele carriers (R_GG/GT_ = −0.198, P_GG/GT_ = 0.209; bottom right in Figure 2). With increasing age, the activation of the brain in the left postcentral region in the TT carriers was significantly increased, while that of the GG/GT carriers was weakened.

### The Effects of rs405509 on Brain FC

According to a meta-analysis (Tseng et al., 2013), we selected some ROIs from executive components of WM to explore WM-related FC and the effects of rs405509 on brain FC. As shown in Table 5, these brain regions included the caudal superior frontal sulcus (SFS), left precentral gyrus (preCG), right precuneus, and some key regions related to WM.

**Table 5:** The key brain regions of working memory performance.

Brain regions	MNI coordinates		
	*X*	*Y*	*Z*
Left caudal SFS	−28	0	58
Left preCG	−42	−4	42
Right caudal SFS	34	6	56
Right IFJ	48	4	38
Right precuneus	12	−66	60
Left SPL/IPS	−22	−64	54
Left precuneus	−12	−72	46
Left IPS/IPL	−34	−38	42

*SFS, superior frontal sulcus; preCG, precentral gyrus; IFJ, inferior frontal junction; SPL, superior parietal lobule; IPS, intraparietal sulcus*.

We used partial correlation analysis to explore the relationship between FC and age in the two groups, and the APOE4 status, gender, and educational level were used as covariates. There was a significant correlation between FC and age in the TT group, but there was no significant correlation in the GG/GT group. These functional connectivities included the left precentral and right caudal SFS (R_TT_ = 0.338, P_TT_ = 0.029) and the right caudal SFS and right precuneus (R_TT_ = 0.342, P_TT_ = 0.027).

## Discussion

Here, we used a large cohort to investigate how the APOE promoter polymorphism influenced brain functional activation and connectivity areas in non-dementia elderly. Interestingly, we found that there was an accelerated age-related increase in functional activation in the left postcentral gyrus in the rs405509 T/T carriers compared with G-allele carriers. Furthermore, the FC between the left postcentral gyrus and some key regions during performance of a WM task, including the right caudal SFS, was modulated by age in different rs405509 genotype groups. In the present study, we found direct evidence to prove that the APOE promoter polymorphism plays a significant role in the neural system.

APOE4 has been studied though the neuroimaging techniques to explore its influence on neurophysiology or neuroanatomy in healthy people. In addition, other polymorphisms in the APOE gene are also important for determining the impact of APOE on the human brain, but they unfortunately have not received enough attention. To our knowledge, we provide the first neuroimaging investigation exploring how APOE promoter polymorphism within influences brain functional activation and connectivity, which means our findings are helpful in clarifying the role of APOE promoter polymorphisms in neural systems. Hence, our study emphasizes the importance of a new genetic factor, i.e., rs405509, to understand the differences in the brain, especially in the elderly without dementia.

### The Effect of rs405509 on Cognitive Aging

APOE promoter polymorphisms are closely related to aging and may have independent mechanisms for normal aging and pathological aging (Rantalainen et al., 2016, 2019). As we found in our study, the results of linear regression analysis showed that the interaction between the rs405509 genotype and age had a significant effect on general cognitive performance and execution function scores. Compared with GG/GT carriers, TT carriers showed a negative correlation between age and MMSE scores. This may be explained partly by the change in brain disconnections. In a recent study by our group, we revealed the modulation effect of the rs405509 genotype on cortical thickness in the parahippocampus and the efficiency of white matter structural networks in the posterior cingulate cortices during nondemented aging (Chen et al., 2015; Shu et al., 2015). In TT carriers, disconnections in TT carriers in brain structure may induce dysfunction. This may be the neural basis for the developing development of cognitive impairments in TT carriers, which was reflected in the reverse correlation between age and general cognition only in the TT and GT/GG genotypes.

### The Effects of rs405509 on Brain Functional Activation

The precentral regions have been shown to have prominent cortical thinning with aging (Salat et al., 2004). Reduced gray matter volume (GMV) has also been observed in some precentral and postcentral cortical regions in patients with AD (Drzezga et al., 2009) and MCI (Wang et al., 2009). The cortical preCG was thinner in APOE4 allele carriers than in non-APOE4 carriers, and this is a later developing region thought to be more susceptible to natural aging processes (Fennema-Notestine et al., 2011).

As one region of interest in the field of cognitive aging, the prefrontal cortex is a critical area because it has been suggested that older adults show worse performance when facing tasks related to executive function, which is supported by this area (Greenwood, 2000). Thus, early age-related changes in this region might result in age-related decreases in performance on executive processing tasks such as WM tasks (Salat et al., 2002). The present data also support this hypothesis. The accelerated age-related increase in functional activation in the left postcentral gyrus was associated with lower cognitive performance.

### The Effects of rs405509 on Brain FC

The daily activities of humans are supported by harmonious relationships between different brain regions (Mesulam, 1998; Buckholtz and Meyer-Lindenberg, 2012). The interactions between brain regions are very important for efficient cognitive function and cognitive impairments (Bernal and Altman, 2010; De Schotten et al., 2011; Buckholtz and Meyer-Lindenberg, 2012). Assessing FC during particular task states makes sense in screening for cognitive disorders. The changes in FC may precede changes in brain activation (Bokde et al., 2006).

Increased FC between the important brain areas occurred early in the course of AD and other cognitive impairments. Recently, some studies have shown that increased local FC strength within the hippocampus was related to lower episodic memory performance in patients with AD (Koch et al., 2015). Even in healthy people, a higher level of interhemispheric FC between the hippocampus was associated with increased age and faster memory decline. Therefore, we suggest that high FC may be a biomarker in subjects with high-risk factors. Meanwhile, our results that FC between the left postcentral gyrus and some key regions during performance of a WM task was modulated by age in different rs405509 genotype groups were consistent with those of previous studies and provided evidence for the hypothesis that high FC may be an important indicator in subjects with high-risk factors.

The mechanism underlying the increased FC in high-risk TT carriers is not known. One reason to explain this finding is that increased brain activation might be attributed to increased deposition of Aβ. Previous studies found a correlation between higher brain activation during memory tasks and Aβ deposition in elderly cognitively healthy subjects and aMCI patients (Huijbers et al., 2015). However, it still needs to be verified whether increased Aβ levels in the brain result in an increase in FC. While the increased Aβ level may evoke abnormal neural activity, this change may not be reflected in FC because FC relies more on the interactions between different brain regions than on local increases in particular brain areas.

There is also another explanation that tries to answer this question from the perspective of brain activity efficiency. This view considers that increased FC represents lower efficiency in brain network activity. According to the dedifferentiation hypothesis, as age increases, brain functional activities lose their specialization in function because of less efficient neural processing (Dennis and Cabeza, 2011). This change in efficiency may require increased FC and more resources to compensate and maintain usual task performance. Unfortunately, the findings in our study were not consistent with this hypothesis, as there was an inverse association between increased FC and performance during the WM task, which might reflect a failed compensatory mechanism. Therefore, more studies in the future are needed to confirm this hypothesis.

### The Potential Pathological Mechanism of rs4055059 on Cognitive Impairments

APOE gene is considered an important risk factor for AD and cognitive impairments. APOE4 carriers show poorer performance in some cognitive domains, such as execution function, than noncarriers (Luck et al., 2015). Consistent with the accelerated decline in cognition, brain structure and function in APOE4 carriers are altered, especially in the medial temporal lobe (Espeseth et al., 2008) and frontal lobe (Fennema-Notestine et al., 2011). Similar to structural differences, differential effects of the APOE genotype on brain function were found in similar brain regions across the life span (Filippini et al., 2011). While the changes in brain activity may depend on task difficulties, this functional activity change is related to cognitive decline (Di Battista et al., 2016).

The APOE promoter rs405509, also known as 2219T/G or Th1/E47cs, is a DNA sequence that acts as a transcriptional factor binding site, and it has been strongly suggested to control APOE expression by influencing parenchymal Aβ deposition (Laws et al., 2003). A recent study also provides evidence for this mechanism, suggesting that the modifying effect of the rs405509 genotype on the association of APOE with risk and age at the onset of AD is due to its influence on the level of APOE protein (Choi et al., 2019). This change in APOE expression is deemed to be related to the physiological mechanism of AD, because of the negative correlation between brain amyloid load and APOE expression level (Lambert et al., 2005).

In addition to the effect of rs405509 on APOE gene expression, the synergistic effect of rs405509 TT and APOE4 was found in AD-related neurodegeneration, which means that the presence of rs40550 may exacerbate the brain changes in high-risk groups such as APOE4 carriers (Choi et al., 2019). In this study, the extent of atrophy in the medial temporal cortex, precuneus, and hippocampus of subjects with TT and APOE4 genotypes was significantly more severe than that of subjects with APOE3 homozygous and TT or APOE4 homozygous and GG genotypes (Choi et al., 2019).The interaction with APOE4 could be taken into consideration. Promoter polymorphisms may increase the dementia risk through the APOE4 pathway (Rantalainen et al., 2019), with expression levels of APOE4 determined by APOE promoter polymorphisms (Lescai et al., 2011). Previous studies have found that rs405509 T homozygosity can modulate the effect of APOE4 on both cognitive performance and brain structure (Ma et al., 2016a,b). TT carriers with APOE4 suffered from a poor cognitive status and reduced GMV in the inferior temporal gyrus and fusiform gyrus, and more importantly, the cognitive and structural changes were significantly correlated (Ma et al., 2016b). This correlation may reflect the neuromechanism of gene interaction at the brain level and contribute to low cognitive function (Ma et al., 2016b). Further studies should place more emphasis on the interaction of rs405509 and APOE4 on pathological aging.

In our current study, although there were more APOE4 carriers in the TT group than in the GT/GG group, the distribution of APOE4 between the two groups was not significantly different (*P* > 0.05). Furthermore, for neuropsychological assessment statistical analysis, a linear regression analysis was used for the neuropsychological test, and APOE4 status was included as a covariate. Thus, the poor cognitive performance in TT carriers was mainly due to the TT genotype. The previous studies also found the independent effect of rs405509 on the brain structural connectivity, which induced the cognitive decline in the nondemented elderly (Chen et al., 2015; Shu et al., 2015; Chang et al., 2017).

The present study had some limitations. First, our study is a cross-sectional analysis, so it is very necessary to validate the present findings by cohort and longitudinal studies. Second, other polymorphisms within the APOE promoter remain ungenotyped in our samples. However, it would be important to the genotype of other polymorphisms and we need to assess the combinatorial effects in the future. Third, we do not know whether genes except this have a role in the rs405509-related effects on task-related FC, which also need a further exploration. Overall, the interpretation of the findings in the current study should be considered in the context of the above limitations.

## Conclusions

In summary, the present study suggested an important role of the rs405509 polymorphism on areas of connectivity in the left postcentral gyrus and FC between the left postcentral gyrus and some key regions during performance of a WM task, with the T/T carriers showing an accelerated age-related increase. The findings in this study suggest the significance of APOE promoter polymorphisms to the neural system.

## Data Availability Statement

The original contributions presented in the study are included in the article, further inquiries can be directed to the corresponding author.

## Ethics Statement

The use of human subjects for this study has been approved by IRB at the Imaging Center for Brain Research at Beijing Normal University. The patients/participants provided their written informed consent to participate in this study.

## Author Contributions

QZ, LW, and XL participated in manuscript preparation and revision. LW, HL, and XL participated in the study design. JS helped in data acquisition. CD and KX carried out MRI data analysis. JZ carried out DNA data analysis. All authors have read and approved the content of the manuscript.

## Conflict of Interest

The authors declare that the research was conducted in the absence of any commercial or financial relationships that could be construed as a potential conflict of interest.

## Footnotes

^1^http://www.fil.ion.ucl.ac.uk/spm
